# Supplementary material for: Modeling glioblastoma heterogeneity as a dynamic network of cell states
Source: Mol Syst Biol. 2021 Sep 16;17(9):e10105. doi: 10.15252/msb.202010105 (PMC8444284; doi:10.15252/msb.202010105)
Supplement: Supplementary file 5 — Source Data for Figure 3 [file MSB-17-e10105-s001.zip › Figure3A_sourcedata/GSEA_3065/hallmarks_state6.GseaPreranked.1623416308702/HALLMARK_G2M_CHECKPOINT.html]

Details for gene set HALLMARK\_G2M\_CHECKPOINT[GSEA]

|  || Dataset | state6 |
| Phenotype | NoPhenotypeAvailable |
| Upregulated in class | na\_neg |
| GeneSet | HALLMARK\_G2M\_CHECKPOINT |
| Enrichment Score (ES) | -0.41558877 |
| Normalized Enrichment Score (NES) | -1.8205199 |
| Nominal p-value | 0.0 |
| FDR q-value | 0.0031003582 |
| FWER p-Value | 0.017 |
Table: GSEA Results Summary

  

Fig 1: Enrichment plot: HALLMARK\_G2M\_CHECKPOINT      
 Profile of the Running ES Score & Positions of GeneSet Members on the Rank Ordered List

  

| PROBE | GENE SYMBOL | GENE\_TITLE | RANK IN GENE LIST | RANK METRIC SCORE | RUNNING ES | CORE ENRICHMENT || 1 | ODC1 |  |  | 222 | 0.169 | -0.0123 | No |
| 2 | ABL1 |  |  | 344 | 0.145 | -0.0147 | No |
| 3 | DR1 |  |  | 346 | 0.145 | -0.0032 | No |
| 4 | FOXN3 |  |  | 369 | 0.140 | 0.0054 | No |
| 5 | GSPT1 |  |  | 455 | 0.127 | 0.0058 | No |
| 6 | HMGN2 |  |  | 679 | 0.108 | -0.0115 | No |
| 7 | TLE3 |  |  | 765 | 0.101 | -0.0133 | No |
| 8 | ATRX |  |  | 861 | 0.094 | -0.0168 | No |
| 9 | EZH2 |  |  | 940 | 0.089 | -0.0187 | No |
| 10 | MYBL2 |  |  | 991 | 0.086 | -0.0177 | No |
| 11 | TOP1 |  |  | 1024 | 0.084 | -0.0146 | No |
| 12 | UPF1 |  |  | 1039 | 0.084 | -0.0096 | No |
| 13 | MARCKS |  |  | 1081 | 0.081 | -0.0078 | No |
| 14 | MT2A |  |  | 1097 | 0.081 | -0.0031 | No |
| 15 | CUL3 |  |  | 1104 | 0.080 | 0.0027 | No |
| 16 | KIF5B |  |  | 1112 | 0.080 | 0.0082 | No |
| 17 | NOLC1 |  |  | 1181 | 0.076 | 0.0065 | No |
| 18 | CUL1 |  |  | 1195 | 0.075 | 0.0110 | No |
| 19 | CCND1 |  |  | 1317 | 0.071 | 0.0026 | No |
| 20 | TNPO2 |  |  | 1376 | 0.068 | 0.0013 | No |
| 21 | SAP30 |  |  | 1409 | 0.067 | 0.0030 | No |
| 22 | AMD1 |  |  | 1460 | 0.066 | 0.0024 | No |
| 23 | CDK4 |  |  | 1490 | 0.064 | 0.0042 | No |
| 24 | STMN1 |  |  | 1649 | 0.058 | -0.0094 | No |
| 25 | RASAL2 |  |  | 1966 | 0.049 | -0.0422 | No |
| 26 | E2F1 |  |  | 2034 | 0.047 | -0.0462 | No |
| 27 | PURA |  |  | 2036 | 0.047 | -0.0426 | No |
| 28 | MYC |  |  | 2081 | 0.045 | -0.0441 | No |
| 29 | JPT1 |  |  | 2094 | 0.045 | -0.0419 | No |
| 30 | CHMP1A |  |  | 2121 | 0.044 | -0.0413 | No |
| 31 | CENPA |  |  | 2199 | 0.042 | -0.0469 | No |
| 32 | TTK |  |  | 2262 | 0.040 | -0.0510 | No |
| 33 | DKC1 |  |  | 2283 | 0.039 | -0.0502 | No |
| 34 | SLC38A1 |  |  | 2301 | 0.039 | -0.0491 | No |
| 35 | PTTG1 |  |  | 2304 | 0.038 | -0.0462 | No |
| 36 | MCM6 |  |  | 2484 | 0.034 | -0.0643 | No |
| 37 | MNAT1 |  |  | 2512 | 0.033 | -0.0648 | No |
| 38 | CDC7 |  |  | 2650 | 0.030 | -0.0783 | No |
| 39 | KATNA1 |  |  | 2681 | 0.029 | -0.0794 | No |
| 40 | ORC5 |  |  | 2687 | 0.029 | -0.0777 | No |
| 41 | CDC25A |  |  | 2768 | 0.027 | -0.0848 | No |
| 42 | CBX1 |  |  | 2863 | 0.025 | -0.0937 | No |
| 43 | TMPO |  |  | 2978 | 0.022 | -0.1051 | No |
| 44 | CKS1B |  |  | 2985 | 0.022 | -0.1041 | No |
| 45 | NUMA1 |  |  | 3025 | 0.021 | -0.1069 | No |
| 46 | NUP50 |  |  | 3103 | 0.020 | -0.1142 | No |
| 47 | NSD2 |  |  | 3106 | 0.020 | -0.1129 | No |
| 48 | PLK1 |  |  | 3116 | 0.020 | -0.1123 | No |
| 49 | DTYMK |  |  | 3271 | 0.016 | -0.1289 | No |
| 50 | YTHDC1 |  |  | 3286 | 0.016 | -0.1293 | No |
| 51 | HNRNPD |  |  | 3359 | 0.014 | -0.1365 | No |
| 52 | HMGB3 |  |  | 3377 | 0.014 | -0.1374 | No |
| 53 | RACGAP1 |  |  | 3432 | 0.012 | -0.1427 | No |
| 54 | SMARCC1 |  |  | 3546 | 0.010 | -0.1550 | No |
| 55 | LIG3 |  |  | 3559 | 0.010 | -0.1555 | No |
| 56 | RAD23B |  |  | 3871 | 0.003 | -0.1914 | No |
| 57 | HIF1A |  |  | 3882 | 0.003 | -0.1923 | No |
| 58 | CDC25B |  |  | 3887 | 0.003 | -0.1926 | No |
| 59 | CDC20 |  |  | 3996 | 0.001 | -0.2050 | No |
| 60 | PAFAH1B1 |  |  | 4151 | -0.002 | -0.2227 | No |
| 61 | NCL |  |  | 4158 | -0.002 | -0.2232 | No |
| 62 | CUL4A |  |  | 4159 | -0.002 | -0.2230 | No |
| 63 | SMAD3 |  |  | 4246 | -0.004 | -0.2327 | No |
| 64 | CDKN1B |  |  | 4475 | -0.008 | -0.2585 | No |
| 65 | SNRPD1 |  |  | 4477 | -0.008 | -0.2580 | No |
| 66 | HMGA1 |  |  | 4523 | -0.009 | -0.2624 | No |
| 67 | HUS1 |  |  | 4532 | -0.009 | -0.2626 | No |
| 68 | POLE |  |  | 4643 | -0.012 | -0.2745 | No |
| 69 | MEIS2 |  |  | 4714 | -0.013 | -0.2816 | No |
| 70 | KMT5A |  |  | 4725 | -0.013 | -0.2817 | No |
| 71 | CCNB2 |  |  | 4736 | -0.013 | -0.2818 | No |
| 72 | DDX39A |  |  | 4809 | -0.014 | -0.2890 | No |
| 73 | HIRA |  |  | 4810 | -0.014 | -0.2878 | No |
| 74 | PML |  |  | 4824 | -0.015 | -0.2881 | No |
| 75 | UCK2 |  |  | 4845 | -0.015 | -0.2892 | No |
| 76 | NOTCH2 |  |  | 4874 | -0.016 | -0.2912 | No |
| 77 | SLC7A5 |  |  | 4959 | -0.017 | -0.2995 | No |
| 78 | SS18 |  |  | 5066 | -0.020 | -0.3103 | No |
| 79 | POLA2 |  |  | 5164 | -0.022 | -0.3198 | No |
| 80 | CTCF |  |  | 5167 | -0.022 | -0.3183 | No |
| 81 | MEIS1 |  |  | 5290 | -0.024 | -0.3305 | No |
| 82 | PDS5B |  |  | 5359 | -0.025 | -0.3364 | No |
| 83 | BUB1 |  |  | 5377 | -0.026 | -0.3363 | No |
| 84 | ARID4A |  |  | 5383 | -0.026 | -0.3348 | No |
| 85 | STAG1 |  |  | 5538 | -0.029 | -0.3503 | No |
| 86 | LMNB1 |  |  | 5545 | -0.029 | -0.3487 | No |
| 87 | MAP3K20 |  |  | 5552 | -0.030 | -0.3470 | No |
| 88 | PRMT5 |  |  | 5662 | -0.032 | -0.3571 | No |
| 89 | BUB3 |  |  | 5807 | -0.034 | -0.3710 | No |
| 90 | MAPK14 |  |  | 5821 | -0.035 | -0.3698 | No |
| 91 | E2F4 |  |  | 5968 | -0.037 | -0.3837 | No |
| 92 | WRN |  |  | 6102 | -0.040 | -0.3959 | No |
| 93 | AURKB |  |  | 6215 | -0.043 | -0.4055 | No |
| 94 | SRSF2 |  |  | 6250 | -0.044 | -0.4060 | No |
| 95 | BARD1 |  |  | 6284 | -0.044 | -0.4062 | No |
| 96 | CDKN3 |  |  | 6291 | -0.044 | -0.4034 | No |
| 97 | TRA2B |  |  | 6303 | -0.045 | -0.4011 | No |
| 98 | CDC45 |  |  | 6326 | -0.045 | -0.4000 | No |
| 99 | GINS2 |  |  | 6461 | -0.049 | -0.4117 | Yes |
| 100 | CUL5 |  |  | 6479 | -0.049 | -0.4097 | Yes |
| 101 | CDC6 |  |  | 6482 | -0.049 | -0.4060 | Yes |
| 102 | RPA2 |  |  | 6501 | -0.050 | -0.4041 | Yes |
| 103 | SRSF10 |  |  | 6530 | -0.050 | -0.4033 | Yes |
| 104 | ODF2 |  |  | 6555 | -0.052 | -0.4020 | Yes |
| 105 | TFDP1 |  |  | 6587 | -0.052 | -0.4014 | Yes |
| 106 | CCNT1 |  |  | 6592 | -0.053 | -0.3976 | Yes |
| 107 | SYNCRIP |  |  | 6600 | -0.053 | -0.3942 | Yes |
| 108 | HMMR |  |  | 6640 | -0.054 | -0.3944 | Yes |
| 109 | CASP8AP2 |  |  | 6650 | -0.054 | -0.3911 | Yes |
| 110 | NUP98 |  |  | 6652 | -0.054 | -0.3869 | Yes |
| 111 | SRSF1 |  |  | 6655 | -0.054 | -0.3828 | Yes |
| 112 | PRIM2 |  |  | 6678 | -0.055 | -0.3810 | Yes |
| 113 | TROAP |  |  | 6707 | -0.056 | -0.3797 | Yes |
| 114 | TGFB1 |  |  | 6712 | -0.056 | -0.3757 | Yes |
| 115 | MKI67 |  |  | 6750 | -0.057 | -0.3754 | Yes |
| 116 | MCM5 |  |  | 6765 | -0.058 | -0.3724 | Yes |
| 117 | CDC27 |  |  | 6787 | -0.058 | -0.3701 | Yes |
| 118 | TENT4A |  |  | 6790 | -0.058 | -0.3657 | Yes |
| 119 | E2F3 |  |  | 6846 | -0.060 | -0.3673 | Yes |
| 120 | PRC1 |  |  | 6918 | -0.062 | -0.3705 | Yes |
| 121 | SLC12A2 |  |  | 6946 | -0.063 | -0.3686 | Yes |
| 122 | ATF5 |  |  | 6981 | -0.064 | -0.3674 | Yes |
| 123 | RAD21 |  |  | 6984 | -0.064 | -0.3625 | Yes |
| 124 | TACC3 |  |  | 7022 | -0.066 | -0.3616 | Yes |
| 125 | POLQ |  |  | 7023 | -0.066 | -0.3563 | Yes |
| 126 | BIRC5 |  |  | 7024 | -0.066 | -0.3511 | Yes |
| 127 | HNRNPU |  |  | 7074 | -0.067 | -0.3514 | Yes |
| 128 | KIF22 |  |  | 7098 | -0.068 | -0.3486 | Yes |
| 129 | G3BP1 |  |  | 7109 | -0.068 | -0.3443 | Yes |
| 130 | CCNF |  |  | 7245 | -0.073 | -0.3542 | Yes |
| 131 | PRPF4B |  |  | 7247 | -0.073 | -0.3485 | Yes |
| 132 | TOP2A |  |  | 7430 | -0.079 | -0.3633 | Yes |
| 133 | KPNB1 |  |  | 7453 | -0.080 | -0.3595 | Yes |
| 134 | HSPA8 |  |  | 7517 | -0.082 | -0.3602 | Yes |
| 135 | SLC7A1 |  |  | 7535 | -0.083 | -0.3555 | Yes |
| 136 | RBM14 |  |  | 7553 | -0.084 | -0.3508 | Yes |
| 137 | MCM3 |  |  | 7597 | -0.086 | -0.3489 | Yes |
| 138 | KIF11 |  |  | 7619 | -0.087 | -0.3444 | Yes |
| 139 | DBF4 |  |  | 7639 | -0.088 | -0.3396 | Yes |
| 140 | SFPQ |  |  | 7661 | -0.089 | -0.3349 | Yes |
| 141 | AURKA |  |  | 7700 | -0.091 | -0.3320 | Yes |
| 142 | NDC80 |  |  | 7734 | -0.093 | -0.3285 | Yes |
| 143 | CDKN2C |  |  | 7743 | -0.093 | -0.3220 | Yes |
| 144 | BRCA2 |  |  | 7772 | -0.094 | -0.3177 | Yes |
| 145 | KNL1 |  |  | 7810 | -0.096 | -0.3143 | Yes |
| 146 | UBE2S |  |  | 7818 | -0.097 | -0.3073 | Yes |
| 147 | CHEK1 |  |  | 7852 | -0.099 | -0.3033 | Yes |
| 148 | PBK |  |  | 7905 | -0.101 | -0.3012 | Yes |
| 149 | CHAF1A |  |  | 7967 | -0.105 | -0.2999 | Yes |
| 150 | DMD |  |  | 7992 | -0.107 | -0.2942 | Yes |
| 151 | KPNA2 |  |  | 8045 | -0.110 | -0.2914 | Yes |
| 152 | SMC1A |  |  | 8047 | -0.110 | -0.2827 | Yes |
| 153 | RBL1 |  |  | 8069 | -0.111 | -0.2763 | Yes |
| 154 | CKS2 |  |  | 8077 | -0.112 | -0.2681 | Yes |
| 155 | MCM2 |  |  | 8099 | -0.114 | -0.2615 | Yes |
| 156 | XPO1 |  |  | 8216 | -0.123 | -0.2651 | Yes |
| 157 | CCNA2 |  |  | 8227 | -0.124 | -0.2563 | Yes |
| 158 | KIF15 |  |  | 8228 | -0.124 | -0.2464 | Yes |
| 159 | LBR |  |  | 8242 | -0.125 | -0.2379 | Yes |
| 160 | KIF4A |  |  | 8300 | -0.132 | -0.2339 | Yes |
| 161 | FBXO5 |  |  | 8310 | -0.133 | -0.2244 | Yes |
| 162 | NASP |  |  | 8318 | -0.134 | -0.2144 | Yes |
| 163 | MTF2 |  |  | 8320 | -0.134 | -0.2038 | Yes |
| 164 | KIF2C |  |  | 8328 | -0.135 | -0.1938 | Yes |
| 165 | CDK1 |  |  | 8351 | -0.139 | -0.1852 | Yes |
| 166 | TPX2 |  |  | 8370 | -0.143 | -0.1759 | Yes |
| 167 | MAD2L1 |  |  | 8427 | -0.150 | -0.1704 | Yes |
| 168 | ILF3 |  |  | 8430 | -0.150 | -0.1587 | Yes |
| 169 | KIF23 |  |  | 8465 | -0.154 | -0.1503 | Yes |
| 170 | ORC6 |  |  | 8482 | -0.157 | -0.1395 | Yes |
| 171 | NUSAP1 |  |  | 8523 | -0.167 | -0.1308 | Yes |
| 172 | KIF20B |  |  | 8576 | -0.180 | -0.1225 | Yes |
| 173 | EWSR1 |  |  | 8626 | -0.201 | -0.1120 | Yes |
| 174 | SMC2 |  |  | 8658 | -0.216 | -0.0984 | Yes |
| 175 | CENPF |  |  | 8703 | -0.240 | -0.0843 | Yes |
| 176 | CENPE |  |  | 8734 | -0.270 | -0.0661 | Yes |
| 177 | UBE2C |  |  | 8748 | -0.286 | -0.0448 | Yes |
| 178 | SMC4 |  |  | 8764 | -0.307 | -0.0220 | Yes |
| 179 | SQLE |  |  | 8767 | -0.319 | 0.0034 | Yes |
Table: GSEA details [plain text format]

  

Fig 2: HALLMARK\_G2M\_CHECKPOINT: Random ES distribution      
 Gene set null distribution of ES for **HALLMARK\_G2M\_CHECKPOINT**
